# Potato Protein Isolate Stimulates Muscle Protein Synthesis at Rest and with Resistance Exercise in Young Women

**DOI:** 10.3390/nu12051235

**Published:** 2020-04-27

**Authors:** Sara Y. Oikawa, Ravninder Bahniwal, Tanya M. Holloway, Changhyun Lim, Jonathan C. McLeod, Chris McGlory, Steven K. Baker, Stuart M. Phillips

**Affiliations:** 1 Department of Kinesiology, McMaster University, Hamilton, ON L8S 4L8, Canada; oikawasy@mcmaster.ca (S.Y.O.); bahniwr@mcmaster.ca (R.B.); limc16@mcmaster.ca (C.L.); mcleoj2@mcmaster.ca (J.C.M.); 2 Faculty of Applied Health & Community Studies, Sheridan College, Brampton, ON L6Y 5H9, Canada; tanya.holloway@sheridancollege.ca; 3 School of Kinesiology and Health Studies, Queens University, Kingston, ON K7L 3N6, Canada; chris.mcglory@queensu.ca; 4 Department of Neurology, Michael G. DeGroote School of Medicine, McMaster University, Hamilton, ON L8S 4K1, Canada; bakersk@mcmaster.ca

**Keywords:** muscle protein synthesis, resistance exercise, potato protein

## Abstract

Skeletal muscle myofibrillar protein synthesis (MPS) increases in response to protein feeding and to resistance exercise (RE), where each stimuli acts synergistically when combined. The efficacy of plant proteins such as potato protein (PP) isolate to stimulate MPS is unknown. We aimed to determine the effects of PP ingestion on daily MPS with and without RE in healthy women. In a single blind, parallel-group design, 24 young women (21 ± 3 years, *n* = 12/group) consumed a weight-maintaining baseline diet containing 0.8 g/kg/d of protein before being randomized to consume either 25 g of PP twice daily (1.6 g/kg/d total protein) or a control diet (CON) (0.8 g/kg/d total protein) for 2 wks. Unilateral RE (~30% of maximal strength to failure) was performed thrice weekly with the opposite limb serving as a non-exercised control (Rest). MPS was measured by deuterated water ingestion at baseline, following supplementation (Rest), and following supplementation + RE (Exercise). Ingestion of PP stimulated MPS by 0.14 ± 0.09 %/d at Rest, and by 0.32 ± 0.14 %/d in the Exercise limb. MPS was significantly elevated by 0.20 ± 0.11 %/d in the Exercise limb in CON (*p* = 0.008). Consuming PP to increase protein intake to levels twice the recommended dietary allowance for protein augmented rates of MPS. Performance of RE stimulated MPS regardless of protein intake. PP is a high-quality, plant-based protein supplement that augments MPS at rest and following RE in healthy young women.

## 1. Introduction

The regulation of skeletal muscle mass in healthy adults is driven largely by contractile activity and protein feeding. Resistance exercise (RE) is a potent anabolic stimulus that results in a marked increase in the rate of muscle protein synthesis (MPS), largely through the activation of a non-insulin-dependent signaling cascade resulting in the upregulation of the kinase activity of the mechanistic target of rapamycin complex 1 (mTORC1) [1]. Recent results [2] have shown that low-load RE (i.e., 30% of 1-repetition maximum [1RM])—when performed to volitional fatigue—is able to stimulate comparable increases in MPS as high-load RE (i.e., 90% of 1RM), in healthy young adults; which has translated to similar increases in muscle mass and strength over a training period [3,4]. Importantly, RE serves to sensitize skeletal muscle to the anabolic effects of protein ingestion, such that rates of MPS are greater than either stimulus independently [5]. 

The MPS response to amino acid feeding can vary markedly depending on the quality of the protein source. Specifically, complete proteins (a protein containing all of the essential amino acids [EAA]), and protein sources higher in leucine, augment rates of MPS to a greater extent than incomplete proteins and those lower in leucine [6,7,8,9,10]. Leucine is able to independently stimulate MPS through its interaction with mTORC1 [11], and thus, is critical for the determination of feeding-induced rates of MPS. Foods such as meat and dairy contain relatively higher quantities of leucine while also containing a full complement of EAAs making them an effective supplemental protein source. However, in comparison to animal-derived proteins (with the exception of isolated soy protein), we know less about how effective other plant-based proteins work to stimulate MPS [12].

Potatoes are an important global food crop with production of potatoes exceeding 374 million metric tons per year [13]. The potato starch industry produces large quantities of potato juice from which high-quality protein can be filtered and extracted. Indeed, as a percent of total protein, potato protein (PP) isolate contains a high relative EAA content [14], and has one of the highest leucine contents of any commercially available plant-based protein isolate [14]. Hence, PP is an attractive plant-derived protein source, similar in quality to animal-derived proteins; however, no study has looked at the efficacy of PP to stimulate MPS alone, or in conjunction with RE, or whether the addition of PP to a diet set at the recommended daily allowance (RDA) would benefit muscle protein anabolism.

The purpose of this study was to examine the effects of PP supplementation to augment total dietary protein intake above the RDA, on rates of MPS with and without RE during 2 weeks of unilateral lower-limb RE training in comparison to the consumption of the RDA alone. As a secondary outcome, we examined changes in expression of proteins associated with the mTORC1 pathway in response to two weeks of supplement ingestion and RE. We hypothesized that, in comparison to the control group consuming only the RDA, the addition of PP would result in augmented rates of MPS and that RE would elevate rates of MPS above those of feeding alone. We also hypothesized that consumption of PP would augment protein expression in the mTORC1 pathway both with feeding alone and when combined with RE.

## 2. Materials and Methods 

### 2.1. Ethical Approval

The study was approved by the Hamilton Integrated Research Ethics Board (HIREB #4969) and conformed to the standards for the use of human subjects in research as outlined by the Canadian Tri-Council Policy on the ethical use of human subjects in research (https://ethics.gc.ca/eng/documents/tcps2-2018-en-interactive-final.pdf). Each participant was informed of the purpose of the study, experimental procedures, and potential risks before written consent was obtained. This trial was registered at clinicaltrials.gov as NCT04302038.

### 2.2. Participants

Twenty-four healthy young women were recruited from the McMaster University campus and the greater Hamilton area in response to local advertisements to participate in this study. Potential participants were screened first by telephone to ensure they were non-smokers, non-diabetic, and between the ages of 18–29 years. Participants were recreationally active but did not meet Canada’s Physical Activity Guidelines (150 min of moderate intensity exercise/week). Exclusion criteria included: participation in >1 resistance exercise session per week; significant loss or gain of body mass in the past 6 months (>2 kg); regular use of: non-steroidal anti-inflammatory drugs; history of chronic disease; current or recently active or remised cancer; infectious disease; and/or gastrointestinal disease; consuming a vegetarian or vegan diet; or any condition or injury that would preclude them from participating in RE. Although we did not control for the menstrual phase of our participants whilst participating in the 21-week intervention, 16 of 24 participants reported contraceptive use (6 in CON and 10 in PP). 

### 2.3. Study Overview

**Figure 1:**
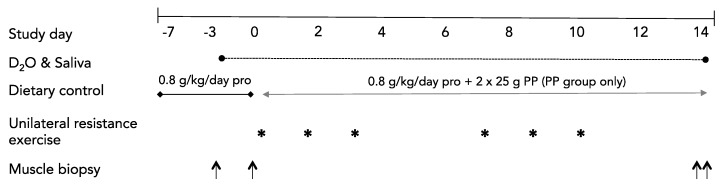
Study schematic. PP, potato protein. * indicates a unilateral resistance exercise session. ↑ indicates one muscle biopsy sample.

An overview of the study is shown in Figure 1. The study was a single blind, parallel group design. Following a controlled dietary run-in period (protocol days −7 to 0), participants were randomized to consume a diet including a twice-daily supplement of PP or to consume a control diet that did not contain a protein supplement (CON). Allocation was concealed from the participants for the duration of the study. After baseline testing and familiarization with all study measures, participants commenced the 21-day protocol during which they consumed a controlled diet with all food provided by the study investigators. Post-testing variables included only skeletal muscle biopsies (one each from the Rest and Exercise legs) and a fasted plasma sample that occurred following an overnight fast on day 14. 

### 2.4. Baseline Testing

Following enrollment, but prior to the commencement of the dietary protocol, participants completed a weighed food record for 3 d (2 week days and 1 weekend day) to assess habitual dietary intake (Nutribase version 11.5, Cybersoft Inc., Phoenix, AZ, USA). Participants also underwent a dual-energy X-ray absorptiometry (DXA) scan (GE-Lunar iDXA; Aymes Medical, Newmarket, ON, Canada) for the determination of total fat- and bone-free [15] body mass (LBM; all CV less than 1.1%). Participants had one of their legs randomly assigned to perform exercise and had the isotonic strength of that leg measured on both (one-repetition maximum, 1RM) a manually loaded leg extension machine (Atlantis Inc., Laval, QC, Canada) and incline leg press machine (Maxim Strength Fitness Equipment, SA, Australia). Following a 5-min warm up on a cycle ergometer, participants were familiarized with the knee extension and leg press machines by performing a single unloaded set for 10 repetitions. Participants rested for 2 min and then began testing for a 1RM. 1RM values were used to calculate the load corresponding to ~ 30% of 1RM to be used for the RE sessions throughout the study protocol. We used a within-subject design to enhance statistical power and to allow for the determination of the resting and exercise responses to a protein supplement within a person [16]. 

### 2.5. Diets

Each participants’ energy requirement was determined with the use of the Harris Benedict prediction equations for basal metabolic rate [17] using height, body mass, activity factors, and age for women. Participants were provided with a weight-maintaining pre-packaged diet for the duration of the trial that provided protein intake at the RDA of 0.8 g/kg/d, on days −7 through −1. At day 0, participants in the PP group were provided with a twice-daily protein supplement (25 g each) mixed with prepared pudding cups which increased total protein intake to 1.6 g/kg/day for 14 days. This was achieved by reducing the proportion of food energy provided from carbohydrates, while the proportion of energy from fat was maintained at ~30% of total energy. Participants in the CON group were provided with a twice daily serving of prepared pudding cups that contained no additional protein (Placebo). Similarly to the PP group, the addition of the placebo supplement (pudding cups with no protein) were added to the diet of individuals in CON by reducing the proportion of food energy provided from carbohydrates. Dietary protein came from a combination of plant- and animal-based protein sources. Participants were prescribed a customized meal plan according to food preferences and food was supplied at the beginning of each week. Participants were also provided with a log in which they were to indicate the percentage of the provided food consumed and were encouraged to consume only the study diet. Any food consumed from outside the provided diet was recorded in the dietary log. Compliance with the prescribed diets and supplements were excellent with subjects consuming 96% ± 2% of what was provided.

### 2.6. Resistance Exercise

Based on the 1RM derived during familiarization, a load calculated to ~30% of the participants 1RM was applied to the leg press and leg extension machines during training. The load was adjusted such that each participant reached volitional fatigue between 20 and 25 repetitions per set. Participants came to the laboratory thrice weekly from days 0–14 to perform unilateral low load RE for a total of 6 RE sessions. Each exercise session began with a brief warm up on a cycle ergometer before the completion of 3 sets of unilateral leg press and 3 sets of unilateral leg extension on the exercised limb. Rest between sets was set at 2 min. 

### 2.7. Supplementation

The PP group consumed a twice-daily supplement of 25 g of potato protein (PP) isolate (Solanic®100F; Avebe, Veendam, The Netherlands). Individual servings of PP or placebo were provided to participants in the PP group every 4 days. All participants were asked to consume the pudding cups within a 5-min period, once in the morning with breakfast and once in the evening 2 h after the dinner meal. The contents of the PP and placebo supplement can be found in Table 1, per serving as provided by the manufacturer. 

### 2.8. Integrated Rates of Muscle Protein Synthesis

Consumption of deuterated water ([^2^H_2_O]; 70% enriched; Cambridge Isotope Laboratories, Andover, MA) was used to label newly synthesized myofibrillar proteins as previously described [18]. Briefly, participants reported to the laboratory in the fasted state on day −3, and after the collection of a saliva sample [17] and rested muscle biopsy, consumed one dose of ^2^H_2_O every 1.5 h, spread over 10.5 h (8 doses total; 0.625 mL/kg of LBM). Additional daily doses (0.625 mL/kg LBM) of ^2^H_2_O were provided to participants to consume each morning following collection of a fasted-state saliva sample. Total body water deuterium enrichment was used as a surrogate of the precursor for plasma alanine labeling [18,19]. Body water enrichment was determined from saliva swabs that were collected by participants between ~0600–0900 each morning. Biopsies, under controlled dietary conditions, at days −3 and 0 (Figure 1) allowed for the determination of baseline (pre-supplementation) rested rates of MPS while days 0 to 14 served as the exercise and nutritional supplementation phases. 

On day 0, participants reported to the laboratory in the fasted state for a rested-state unilateral biopsy. On the morning of day 14, participants returned to the laboratory following an overnight fast for the collection of bilateral muscle biopsies for the determination of integrated rates of MPS (MPS with the integration of all periods of feeding, fasting, exercise, and rest). 

All biopsies were taken following administration of 1% xylocaine local anesthesia with the use of a 5 mm Bergström needle that was adapted for manual suction. Muscle tissue samples were freed from any visible connective and adipose tissue, rapidly frozen in liquid nitrogen and stored at −80 °C for further analysis.

### 2.9. Analytical Methods

Muscle samples (~30–50 mg) were homogenized to yield the myofibrillar protein fraction. Samples were homogenized on ice in buffer (10 μL/mg 25 mM Tris 0.5% v/v Triton X-100 and protease/phosphatase inhibitor cocktail tablets [Complete Protease inhibitor Mini-Tabs; Roche, and PhosSTOP; Roche Applied Science]) and centrifuged at 1500× *g* for 10 min at 4 °C. The supernatant was removed for the protein expression analysis and the pellet was retained. For the measurement of MPS, the myofibrillar protein pellet was solubilized and centrifuged as previously described [20], and the supernatant containing the myofibrillar proteins was collected. Myofibrillar proteins were precipitated in 1 mL of 1 M perchloric acid, the supernatant discarded, and the fraction was washed twice with 70% ethanol. The myofibrillar protein-enriched pellets were hydrolyzed in 0.5 M HCl at 110 °C for 72 h to release their respective amino acids. Protein bound amino acids were purified by ion exchange chromatography on Dowex H^+^ resin. Myofibrillar ^2^H-alanine enrichments were determined as previously described [18].

### 2.10. Western Blotting

Expressions of intracellular signaling proteins were assessed by Western blotting. Following homogenization for integrated MPS, total protein concentration of the sarcoplasmic fraction was determined by using a bicinchoninic acid assay (Thermo Fisher Scientific, Waltham, MA, USA). Working samples of equal concentration were prepared in 4X Laemmli buffer. Equal amounts of protein (10 μg) from each sample were run on 4%–15% Criterion TGX Stain-Free protein gels (Bio-Rad, Hercules, CA, USA) at 200 V for 45 min. A protein ladder (Precision Plus Protein Standard, Bio-Rad) and a calibration curve were run on every gel [21]. Proteins were then transferred to nitrocellulose membranes and were blocked for 1 h in 5% bovine serum albumin. Transfer was visually checked with ultraviolet (UV) activation of the gel as well as the membrane pre- and post-transfer (ChemiDoc MP Imaging System; Bio-Rad). Membranes were then exposed for 12 h at 4 °C to primary antibodies after which they were washed in Tris-buffered saline and Tween 20 (TBS-T, Millipore Sigma) and incubated in anti-rabbit IgG (Immunoglobin G) conjugates with horseradish peroxidase secondary antibodies (1:3000; 7074S; Cell signaling Technology, Dancers, MA, USA) for 1 h at room temperature. Signals were detected by using chemiluminescence solution (Clarity Western ECL substrate, Bio-Rad), and bands were quantified by using Image Lab 6.0.1 (Image Lab Software for Mac Version 6.0.1, Hercules, CA, USA). Protein content was normalized using the calibration curve obtained from each gel The following antibodies used were purchased from Cell Signaling Technology (Danvers, MA, USA): t-4E-BP1 (1:1000; 9644S), mechanistic target of rapamycin (t-mTOR; 1:1000; 2972S), protein kinase B (t-AKT; 1:1000; 4691S), ribosomal protein S6 (t-S6; 1:1000; 2217L) and p70 S6 kinase 1 (t-p70S6K1; 1:1000; 9202L). Analyses of candidate protein expressions were conducted at Baseline, Rest, and Exercise on day 14. 

### 2.11. Saliva Sample Analusis

Saliva samples were analyzed for ^2^H enrichment by cavity ring-down spectroscopy using a liquid isotope analyzer (Picarro L2130-*I* analyzer, Picarro, Santa Clara, CA, USA) with an automated injection system. The water phase of saliva was injected six times per sample and the average of the last three measurements were used for data analysis (coefficient of variation ≤0.8%). Standards were measured before and after each participant. The ^2^H isotopic enrichments for muscle and saliva initially expressed as δ^2^H ‰ relative to Vienna Standard Mean Ocean Water (VSMOW) and were converted to atom percent excess (APE) using standard equations [18]. 

### 2.12. Calculations

The fractional synthetic rate of myofibrillar proteins determined %/d for [^2^H]-alanine using the precursor–product equation as previously described [22,23,24]. 

### 2.13. Statistics

Dietary variables (fat, carbohydrate, and protein intake) were compared using an independent sample *t-*test. MPS and western blotting analyses were compared using a 2-way analysis of variance (ANOVA) with between-persons (group: PP versus CON) and within-person (condition: Baseline, feeding [Rest], feeding + exercise [Exercise]) factors. All significant interactions from the ANOVA analysis were further tested by a post-hoc using Tukey’s post hoc test. Significance was set at *p* ≤ 0.05. All statistical analyses were completed using SPSS (IBM SPSS Statistics for Mac, version 21; IBM Corp., Armonk, NY, USA). Graphical representations of data are as individual values with the horizontal line represents the mean, and the whiskers representing the 95% confidence interval. All other values, unless otherwise noted, are expressed as means ± standard deviation. 

## 3. Results

### 3.1. Participants’ Characteristics

Participant characteristics are presented in Table 2. There were no significant differences at baseline between groups with the exception of leg press 1RM per kg of body mass which was higher in the PP group. 

### 3.2. Dietary Intake

Dietary intake variables are shown in Table 3. There were no differences in total kcals or percentage of fat intake between the PP and CON groups (*p* > 0.05). Protein intake was significantly greater in the PP group during the 2-week intervention period (*p* < 0.0001). 

### 3.3. Integrated Myofibrillar Protein Synthesis (MPS)

**Figure 2:**
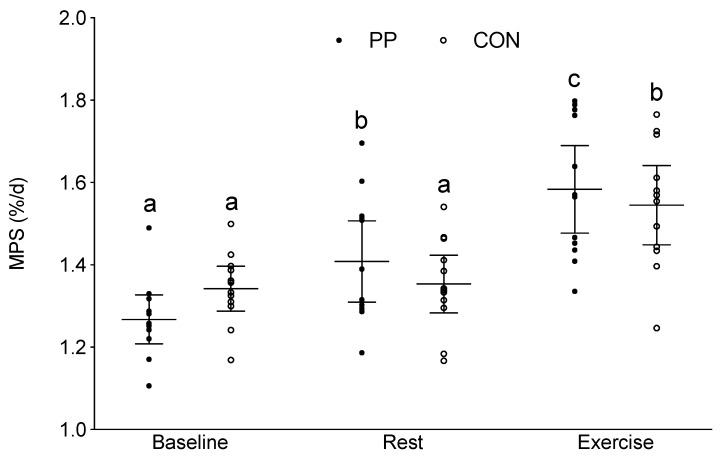
Integrated myofibrillar protein synthesis (%/day) at Baseline, and at Rest and with Exercise during the supplementation period. The horizontal line represents the mean, and the whiskers represent the 95% confidence interval. Means that do not share a letter are significantly different within the same group, *p* = 0.008. FSR, fractional synthetic rate; PP, potato protein supplemented group; and CON, control diet group.

In the PP group, additional protein ingestion increased MPS above Baseline by 0.14 ± 0.09 %/d at Rest, and increased MPS by 0.18 ± 0.01 %/d with Exercise above Rest (95% confidence interval (CI): 1.37, 1.47; η^2^ = 0.196, *p* = 0.008). In the CON group, MPS was only significantly elevated above Baseline with Exercise (0.20 ± 0.11 %/d) and was not significantly elevated from Baseline at Rest (0.01 ± 0.04 %/d) (Figure 2). There were no significant differences between groups at any time point.

### 3.4. Muscle Signaling Protein Content

**Figure 3:**
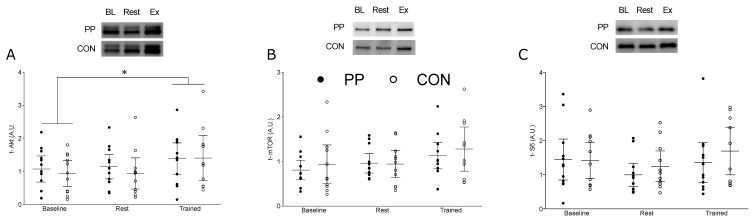
Total protein expression at Baseline (Day 0), and at Rest and Exercise during the supplementation period at Day 14. (**A**) Total protein expression of Akt; (**B**) total protein expression of mTOR, and (**C**) total protein expression of s6. The horizontal line represents the mean, and the whiskers represent the 95% confidence interval. * Indicates significantly greater than Baseline, *p* < 0.0001. PP, potato protein supplemented group; CON, control diet group.

In response to Exercise, t-Akt was significantly increased compared to baseline (95% CI: 0.84, 1.44, η^2^ = 0.009, *p* < 0.0001); however, t-Akt was not elevated above baseline at Rest (Figure 3A). ANOVA revealed a significant main effect of time for t-mTOR (95% CI: 0.81, 1.19, η^2^ = 0.007, *p* = 0.004) (Figure 3B) and t-s6 (95% CI: 1.06, 1.7, η^2^ = 0.027, *p* = 0.041) (Figure 3C) however post-hoc analyses did not yield a significant difference between any measurement points. There were no significant differences between time points for t-p70S6K (95% CI: 0.90, 1.06, η^2^ = 0.068, *p* = 0.85) and t-4EBP1 (95% CI: 0.97, 1.48, η^2^ = 0.032, *p* = 0.955). 

## 4. Discussion

We report, for the first time, that the ingestion of PP isolate resulted in increased rates of MPS when consumed at levels twice the RDA for protein in healthy young women. Our findings highlight that PP is effective in stimulating rates of MPS with supplementation alone and that this effect is enhanced when feeding with PP isolate is combined with RE. We note, however, that despite an apparently greater stimulation of MPS with PP with Exercise (12.7% ± 7.8% greater than rest), rates were similar to that seen in CON with Exercise (14.2% ± 8.7% greater than rest), which highlights the relative potency of RE as a stimulus for MPS compared to PP ingestion. 

Dietary proteins are scored for their quality using the protein digestibility corrected amino acid score (PDCAAS) or by the digestible indispensable amino acid score (DIAAS) [25]. Both the PDCAAS and the DIAAS determine protein quality by the EAA content and the digestibility of the protein. Following protein ingestion, it is the large postprandial hyperaminoacidemia, specifically EAA and leucine in particular, that drive the stimulation of MPS [26]. Of the EAA, leucine has been shown to independently stimulate rates of MPS and as such, protein sources higher in leucine content typically elicit a greater stimulation of MPS [8,27,28]. PP isolate has a high DIAAS score of 0.94 (L. Herreman, Avebe, personal communication) and has a relatively high leucine content (~8.3% of total amino acids), among the highest of all plant based protein isolates [14]. Previously, we have shown that consumption of beverages higher in leucine resulted in augmented rates of MPS in comparison to the consumption of beverages containing low quantities of leucine at rest in healthy young men [7]. Thus, the significant increase in MPS following PP ingestion in comparison to the Baseline diet in the current study population might be expected given the high protein quality score of PP isolate and its leucine content. This rise in MPS (Figure 2) also serves to highlight that the protein RDA did not provide sufficient protein to fully stimulate MPS.

Performance of RE serves to sensitize skeletal muscle to the anabolic effects of protein ingestion, such that rates of MPS are further enhanced when protein is consumed in temporal proximity to performance of RE [8,29]. Our finding that high repetition RE and supplementation with PP increased rates of MPS above the consumption of PP isolate alone is in line with previous acute measurements from our laboratory in healthy young men [8,29] as well as previous investigations measuring integrated rates of MPS in healthy older women [9,30,31]. Burd et al. reported that both higher repetition (lower weight) and lower repetition (higher weight) RE when performed to volitional fatigue and combined with protein ingestion, facilitated elevations in MPS and the Akt/mTOR pathway for 24 h [29]. Although integrated rates of MPS (the measurement of MPS including all periods of fasting, feeding, rest and exercise) do not allow for the measurement of acute post-exercise elevations in MPS, we can hypothesize that given that the high repetition RE performed was to volitional fatigue, and that the PP supplement was consumed within the 24 h “anabolic window” [32], that rates of MPS would have been high, if not maximal. Unsurprisingly, we did not see an increase in MPS at Rest in the CON group (consuming only the RDA for protein), however in young women, RE was able to augment rates of MPS above those seen at Baseline and Rest. 

We also aimed to investigate the effect of PP on skeletal muscle protein kinetics through the examination of total content of the key proteins involved in the mTORC1 pathway and its downstream effectors. Although we did not find any interaction between supplemental groups, there was an increase in total Akt in the exercised limb that was significantly elevated above rest and feeding alone with no difference between groups. In combination with the short duration of our exercise and supplementation intervention, our biopsy time points were chosen for the determination of our primary outcome measure (MPS). Thus, we hypothesize that 72 h after exercise cessation was past the opportune window to see augmentations in total protein of the mTOR pathway.

Typically, plant-based proteins are of lower protein quality—based on PDCAAS or DIAAS—than animal-derived proteins and in most cases isolated proteins follow this axiom [12]. Work from our laboratory [33], and others [34], has shown that despite a high protein quality score (0.98 and 0.9 for the PDCAAS and DIAAS, respectively), the consumption of a twice-daily soy protein supplement resulted in smaller increases in type II muscle fiber area and fat-and bone-free mass in comparison to a group consuming fluid milk. We note that the PDCASS and DIAAS scores for milk and soy-derived proteins are different but still high (1.00 and 1.14 for the PDCAAS and DIAAS, respectively [33]) and yet we observed differences in gains in lean mass, in line with what others have observed using isolated whey and soy proteins [34]. Interestingly, PP isolate is a unique and largely unexamined supplemental protein source that when separated from starch and anti-nutritional components, such as dietary fiber, is a high-quality protein, comparable in quality to animal based proteins and with the potential to induce comparable anabolic effects. Future research should focus on the comparison of PP supplementation to meat or dairy-based proteins to further elucidate its anabolic potential.

Our study is the first to our knowledge to examine the effects of PP supplementation in any capacity in humans. Our findings of augmented rates of integrated MPS both with feeding alone and when combined with RE demonstrate the anabolic effects of PP and highlight its potential usage in plant-based diets to meet protein needs. However, our study is not without limitations. In particular, we did not measure the acute plasma amino acid response to PP ingestion and, therefore, cannot comment on the postprandial rise in EAAs (leucine in particular) that are likely underpinning the elevated rates of MPS with PP supplement ingestion. Furthermore, as mentioned, our supplementation and exercise period consisted of only 14 days and thus future investigations should aim to determine the efficacy of prolonged PP ingestion to induce RE-induced muscle hypertrophy. 

## 5. Conclusions

In summary, we report that the consumption of a twice-daily supplement of PP was able to augment rates of MPS above a baseline diet containing the RDA for protein (0.8 g/kg/day), and that rates of MPS were augmented above the ingestion of protein alone when combined with high-repetition RE. However, we did not observe a difference between groups at any time point. Given recent shifts in dietary recommendations and personal preferences, the need for higher-quality protein sources are imperative in order to support nutritional needs. Importantly, when selecting plant-derived protein sources to augment dietary protein intake, sources should contain all EAAs in sufficient quantities and ideally contain adequate leucine to support muscle health, especially with increasing age. PP isolate is a unique and efficacious plant-based protein that should be considered for use to increase total dietary protein intake above the RDA and successfully augment high-quality dietary protein intake. 

## Figures and Tables

**Table 1:** Amino acid composition of the potato protein (PP) supplement (25 g/serving) and energy intake of PP and control (CON) supplements.

Amino acid (g)	PP	CON
Isoleucine	1.4	-
Leucine	2.5	-
Valine	1.6	-
Lysine	1.8	-
Methionine	0.6	-
Phenylalanine	1.6	-
Threonine	1.4	-
Tryptophan	0.3	-
Histidine	0.6	-
Cysteine	0.4	-
Tyrosine	1.3	-
Arginine	1.3	-
Alanine	1.2	-
Aspartic Acid + Asparagine	2.9	-
Glutamic acid + Glutamine	2.8	-
Glycine	1.2	-
Proline	1.2	-
Serine	1.3	-
ΣEAA	11.6	-
ΣNEAA	13.5	-
Total energy per supplement (kcal)	300	200
Carbohydrate (g)	19	19

WHO, World Health Organization; EAA, essential amino acids; NEAA, non-essential amino acids. The amino acid requirements for adults over the age of 19 (in mg/kg/day) as designated by the World Health Organization are: isoleucine, 20; leucine, 39; valine, 26; lysine, 30; methionine 10.4; phenylalanine, 25; threonine, 15; tryptophan, 4; and histidine, 10. Total non-essential amino acid requirement are 184 mg/kg/d.

**Table 2:** Participants’ characteristics.

	PP (*n* = 12)	CON (*n* = 12)	*p*
Age (y)	20 ± 3	21 ± 3	0.41
Height (m)	1.68 ± 0.44	1.64 ± 0.79	0.10
Body mass (kg)	64.4 ± 8.2	61.9 ± 11.4	0.55
BMI (kg/m^2^)	22.8 ± 2.3	23.1 ± 3.0	0.78
Body fat, (%)	28.4 ± 5.9	31.5 ± 5.5	0.20
LBM (kg)	42.9 ± 3.5	39.8 ± 6.6	0.16
Knee extensor 1RM (kg)	44 ± 8	36 ± 7	0.08
Knee extensor 1RM, per kg body mass (kg)	0.69 ± 0.13	0.58 ± 0.07	0.08
Leg press 1RM (kg)	86 ± 29	68 ± 18	0.07
Leg press 1RM, per kg body mass (kg)	1.34 ± 0.42	1.12 ± 0.33	0.02

Values are means ± SD. BMI, body mass index; CON, control group; LBM, lean body mass; PP, potato protein supplemented group; 1RM, one-repetition maximum.

**Table 3:** Dietary intake during the intervention.

	PP (*n* = 12)	CON (*n* = 12)	*p*
Protein (g/kg/d)			
Baseline	0.80 ± 0.01	0.80 ± 0.01	0.68
Supplementation	1.59 ± 0.09 *	0.80 ± 0.01	<0.001
Non-supplemental protein (% of total)	51 ± 3	100 ± 0	
Energy (kcal/kg)			
Supplementation	34 ± 3	33 ± 5	0.34
Carbohydrates (g/kg)			
Supplementation	4.4 ± 0.5	5.0 ± 0.8	0.08
Fat (g/kg)			
Supplementation	1.2 ± 0.1	1.0 ± 0.3	0.17

Values are means ± SD. Baseline (days −7 to −1), Supplementation (days 0–14). * Significantly greater than CON during supplementation, *p* < 0.05.
